# Year 2021 – the year of hope and health care workers

**DOI:** 10.1080/02813432.2021.1922830

**Published:** 2021-07-20

**Authors:** Päivi Korhonen

**Affiliations:** Professor of General Practice Turku University and Turku University Hospital, Turku, Finland

Learn from yesterday, live for today, hope for tomorrow.

Albert Einstein

I am writing this on the 30th of January 2021. Exactly one year ago the World Health Organization (WHO) declared the coronavirus outbreak a public health emergency of international concern, the highest level of alarm under international law. To date, there have been more than 100 million confirmed cases of coronavirus disease 2019 (COVID-19) including over 2 million deaths [1] and countless numbers of grief and sorrow. But – at the same time – we now have new hope that vaccination will put an end to this global tragedy.

Yet here we are amidst the greatest health care crisis of our generation. Clinicians all over the world take care for and cure the patients infected by COVID-19. Health care professionals are facing huge emotional stressors including grief from seeing patients die, fears of getting infected by the virus and infecting their close ones. Many non-clinician experts, epidemiologists, virologists, and immunologists have provided a surge of information to lead us through this catastrophe. The pace at which the clinicians have adapted to this new knowledge is astonishing. Digital communication platforms have helped us to keep a distance from each other, but these new types of software may also turn out to be additional sources of stress.

On top of this all, primary health care has to face the physical, mental and socioeconomic consequences of the pandemic, which certainly will affect peoplés needs and access to care in the years to come.

In the light of all this, it seems correct and reasonable that WHO has designated 2021 the International Year of Health and Care Workers ‘in recognition of their dedication to providing care during and despite the COVID-19 pandemic’ [2]. Beyond offering praise and applause, WHO aims to draw attention of the member states to the need for greater investments in health and care workforce. In Nordic countries, governments should above all address the shortfall in primary care personnel. Even before the pandemic, general practitioners (GPs) got increased work responsibilities [3], and a substantial proportion of GPs reported symptoms of burnout and decreased job satisfaction [4–6]. A recent Norwegian study about motivational experiences of the GPs concluded that GPs should be able to expect that authorities share their strong sense of moral duty and improve the structural frames of their work environment [7]. Attending this goal is now on the agenda of WHO, too.

We all should pay more attention to our work well-being and supporting our colleagues. GPs too often feel that physical and emotional exhaustion is part of the job. We too often share the ethos that vulnerability is a sign of weakness. This is especially strenuous for the young physicians. In Finland, participation in a Balint group is nowadays recommended during the specialty training in general practice. Clinicians tend to accept support from colleagues who understand the stressors of the occupation [8]. Thus, we should more often ask our colleagues: ‘How are you holding up?’

Year 2021 *must* be the turning point of the COVID-19 pandemic, it restores all hope and faith for the good times to return. Hopefully year 2021 can promise even more; faith for true science, and better well-being for health care workers.
